# Bioactive Interpenetrating Hydrogel Networks Based on 2-Hydroxyethyl Methacrylate and Gelatin Intertwined with Alginate and Dopped with Apatite as Scaffolding Biomaterials

**DOI:** 10.3390/polym14153112

**Published:** 2022-07-30

**Authors:** Marija M. Babić Radić, Vuk V. Filipović, Jovana S. Vuković, Marija Vukomanović, Marina Rubert, Sandra Hofmann, Ralph Müller, Simonida Lj. Tomić

**Affiliations:** 1 University of Belgrade, Faculty of Technology and Metallurgy, Karnegijeva 4, 11000 Belgrade, Serbia; mbabic@tmf.bg.ac.rs (M.M.B.R.); jjovasevic@tmf.bg.ac.rs (J.S.V.); 2 University of Belgrade, Institute for Chemistry, Technology and Metallurgy, Njegoseva 12, 11000 Belgrade, Serbia; vukan87@yahoo.com; 3 Jožef Stefan Institute, Advanced Materials Department, Jamova Cesta 39, 1000 Ljubljana, Slovenia; marija.vukomanovic@ijs.si; 4 Institute for Biomechanics, ETH Zurich, Leopold-Ruzicka-Weg 4, 8093 Zurich, Switzerland; marinarubert@gmail.com (M.R.); s.hofmann@tue.nl (S.H.); ram@ethz.ch (R.M.); 5 Department of Biomedical Engineering and Institute for Complex Molecular Systems, Eindhoven University of Technology, P.O. Box 513, 5600 MB Eindhoven, The Netherlands

**Keywords:** 2-hydroxyethyl methacrylate, gelatin, alginate, hydroxyapatite, hydrogel scaffolding biomaterials, biocompatibility, tissue regeneration engineering

## Abstract

Our goal was to create bioimitated scaffolding materials for biomedical purposes. The guiding idea was that we used an interpenetrating structural hierarchy of natural extracellular matrix as a “pattern” to design hydrogel scaffolds that show favorable properties for tissue regeneration. Polymeric hydrogel scaffolds are made in a simple, environmentally friendly way without additional functionalization. Gelatin and 2-hydroxyethyl methacrylate were selected to prepare interpenetrating polymeric networks and linear alginate chains were added as an interpenetrant to study their influence on the scaffold’s functionalities. Cryogelation and porogenation methods were used to obtain the designed scaffolding biomaterials. The scaffold’s structural, morphological, and mechanical properties, in vitro degradation, and cell viability properties were assessed to study the effects of the preparation method and alginate loading. Apatite as an inorganic agent was incorporated into cryogelated scaffolds to perform an extensive biological assay. Cryogelated scaffolds possess superior functionalities essential for tissue regeneration: fully hydrophilicity, degradability and mechanical features (2.08–9.75 MPa), and an optimal LDH activity. Furthermore, cryogelated scaffolds loaded with apatite showed good cell adhesion capacity, biocompatibility, and non-toxic behavior. All scaffolds performed equally in terms of metabolic activity and osteoconductivity. Cryogelated scaffolds with/without HAp could represent a new advance to promote osteoconductivity and enhance hard tissue repair. The obtained series of scaffolding biomaterials described here can provide a wide range of potential applications in the area of biomedical engineering.

## 1. Introduction

There is a growing medical need for the development of multifunctional biomaterials that will meet complex requirements such as biodegradation, biocompatibility, pore size and porosity extent, mechanical characteristics, surface characteristics, and vascularization which are prerequisites for longevity and quality of life [1,2,3]. We used the “path” to design a scaffolding biomaterial that operates as a “replica” of the natural extracellular matrix (ECM) and leads to the development of a modern scaffolding platform for cell vivification and new living. The hierarchical levels of the natural ECM network architecture provide mechanical support and a biologically interactive microenvironment for cell and tissue integration. The interpenetrated network (IPN) structure of native ECM consists of crosslinked proteins interlocked with biomacromolecules [4]. The scaffold creates the environment where the most ideal conditions can be achieved for the growth of new tissue to take place in the least disturbed milieu and promote natural regenerative processes [5]. Many elements of our body contain hydrogels in the form of the extracellular matrix, collagen, mucous, gelatin, cartilage, meniscus, epidermis, vitreous humour, and tendons [4]. Designed hydrogel scaffolds containing these components can be considered as medical devices that could guide the regeneration process.

The 3D structure and hydrophilicity of polymeric hydrogels enable them to hold a significant quantity of water/biological fluids [6,7,8,9,10,11,12]. The dynamic crosslinked polymeric structure builds hydrogels, thus maintaining the integrity of the hydrogel network and therefore remains insoluble in aqueous media. The high water content in the hydrogel scaffold contributes to nutrient diffusion. Such hydrogels also possess properties such as elasticity and flexibility similar to the native ECM, while also providing structural and biochemical sustenance for the cells. The various functional groups involved in the composition of polymeric hydrogel scaffolds can interact with cells and tissues to improve the effectiveness of the regeneration process. This determines how a tissue originates and functions, making hydrogels crucial materials for tissue regeneration engineering [13,14,15,16,17,18].

There are many approaches in the design and methodology of making scaffolding constructs for tissue regeneration and each has advantages and disadvantages [19]. Research related to the synthesis and testing of scaffolding biomaterials based on versatile polymers [20,21] and especially alginate and gelatin for use in hard tissue regeneration is based on functionalized polymers, such as oxidized alginate or methacrylated gelatin, with the addition of inorganic components based on various apatites [22,23,24,25,26,27,28,29,30,31,32,33]. Alginates, natural multifunctional polymers, are attractive biomaterials for biomedical and pharmaceutical uses due to versatile biological activities. Alginates are linear anionic polysaccharides derived from brown algae cell walls (Macrocystis pyrifera, Laminaria hyperborea, Ascophyllum nodosum) [34]. Their physicochemical properties have been studied in detail since their discovery more than a hundred years ago [35,36,37]. They were shown to be biocompatible both in vitro and in vivo and biodegradable in the human body [38,39,40,41]. Gelatin is obtained by denaturation or partial hydrolysis of collagen and is a major biomacromolecule component of the natural ECM of soft and hard tissues [42,43,44,45,46,47,48]. Gelatin possesses protease cleavage sites and cell-interactive functional groups, especially, the Arg-Gly-Asp sequence present in adhesion proteins of the natural ECM [42,43,44,45,46,47,48], which improves cell adhesion. Gelatin-based scaffolds can be easily degraded by proteases [48], which is essential to make space for the deposition of newly formed ECM by cells. When used as a component in hydrogel scaffold composition, gelatin improves suitability for three-dimensional biomedical engineering applications [32,42,43,44,45,46,47,48]. Poly(2-hydroxyethyl methacrylate) (PHEMA) is an outstanding synthetic, hydrophilic polymer. PHEMA-based biomaterials have been successfully applied in ophthalmology, wound dressings, hemodialysis membranes, controlled drug release systems, and devices for soft tissue reconstructive surgery [49,50,51,52]. These applications were enabled by favorable properties, such as hydrophilicity, swelling, biocompatibility, inertness, and tissue-like mechanical characteristics [53,54,55,56,57]. Apatites are known as one of the most remarkable inorganic materials in hard tissue replacement and reconstruction [58]. The similarity of calcium phosphate compounds to the mineral phase of tissues and their biocompatibility has led to widespread applications in tissue regeneration [59].

In our study, we have applied an efficient, environmentally friendly and simple approach to produce scaffolding biomaterials for tissue regeneration, using gelatin and alginate without functionalization. We have designed interpenetrating polymeric networks based on 2-hydroxyethyl methacrylate and gelatin, with the addition of alginate as an interpenetrant using model similarity interpenetrating component hierarchy of natural extracellular matrix. Each of these components has its advantages and was used to improve the overall scaffolding properties—gelatin and alginate similarity with ECM contribute to biological activity, and PHEMA improved mechanical strength. We evaluated the scaffold’s functionalities important for tissue regeneration application. Extensive biological assays were performed including apatite as an inorganic agent to assess the bioactive potential of the obtained hydrogel scaffolds.

## 2. Materials and Methods

### 2.1. Materials

2-Hydroxyethyl methacrylate monomer (HEMA), gelatin from porcine skin (G, Type B), sodium alginate (A), ethyleneglycol dimethacrylate (EGDMA), as well as crosslinker 1-ethyl-3-(3-dimethyl aminopropyl) carbodiimide hydrochloride (EDC), potassium persulfate (PPS), N,N,N′,N′-tetramethylene diamine (TEMED), Pluronic F-172, sodium bicarbonate (NaHCO_3_) were purchased from Sigma-Aldrich, St. Louis, MO, USA. RPMI-1640 medium and supplements for cell proliferation as well as 3-(4,5-dimethylthiazol-2-yl)-2,5-diphenyltetrazolium bromide (MTT) reduction assay components were purchased from Sigma-Aldrich. All syntheses were performed in deionized water. Materials used for the hydroxyapatite synthesis and doping were calcium nitrate pentahydrate (Ca(NO_3_)_2_×5H_2_O) (Sigma-Aldrich), magnesium nitrate hexahydrate (Mg(NO_3_)_2_×6H_2_O), strontium nitrate (Sr(NO_3_)_2_), gallium nitrate hydrate (Ga(NO_3_)_3_×H_2_O), zinc nitrate hexahydrate (Zn(NO_3_)_2_×6H_2_O), ammonium dihydrogen phosphate (NH_4_H_2_PO_4_) were obtained from Sigma-Aldrich, and urea ((NH_2_)_2_CO) was purchased from Alfa Aesar (Haverhill, MA, USA), and all were of analytical grade. All experiments were performed using lab-produced, ultra-distilled water.

### 2.2. Hydrogel Syntheses

Cryogels made of interpenetrating hydrogel networks consisting of HEMA and gelatin were synthesized using free-radical polymerization/crosslinking at −18 °C for 24 h. Gelatin/HEMA = 0.2/0.8 (weight ratio) was dissolved in deionized water and stirred at room temperature. The next step was the addition of agents for cryogelation (Table 1). The reaction mixture was transferred to a Petri dish and placed to perform cryogelation.

Cryogels made of semi-interpenetrating hydrogel networks consisting of HEMA and gelatin were synthesized using free-radical polymerization/crosslinking, using alginate as interpenetrant, at −18 °C for 24 h (Table 1). Gelatin/HEMA/alginate = 0.1/0.8/0.1 were dissolved in deionized water and stirred at room temperature. The next step was the addition of agents for cryogelation (Table 1). The reaction mixture was transferred to a Petri dish and placed to perform cryogelation. Cryogel samples were washed with deionized water for 7 days. Water was changed daily. Swollen gels were frozen and freeze-dried. Cryogels marks are in Table 1.

Porogenated interpenetrating hydrogel networks consisting of HEMA and gelatin (gelatin/HEMA = 0.2/0.8) were synthesized using free-radical polymerization/crosslinking, in water at 63 °C, using NaHCO_3_ as a pore-forming agent and Pluronic F172 as foam stabilizer (Table 1). Crosslinking agents were BIS and EDC for HEMA and gelatin, respectively.

Porogenated semi-interpenetrating hydrogel networks made of HEMA and gelatin were synthesized using free-radical polymerization/crosslinking, and alginate as interpenetrant (gelatin/HEMA/alginate = 0.1/0.8/0.1), in a mixture of water at 63 °C, using NaHCO_3_ as a pore-forming agent and Pluronic F172 as foam stabilizer (Table 1). Porogenated samples were washed with deionized water for 7 days. Water was changed daily. Swollen porogenated samples were frozen and freeze-dried. Porogenated samples were designated as in Table 1.

Hydroxyapatite doped with metals was incorporated (5% of total hydrogel disk weight) during hydrogel synthesis, while the reaction mixture was vigorously stirred to achieve the optimal distribution of HAp particles. Hydrogel scaffolds loaded with HAp were designated as HG_cryo_/HAp and HGA_cryo_/HAp.

### 2.3. Hydrogel Scaffold Characterization

#### 2.3.1. Fourier Transform Infrared Spectroscopy (FTIR)

Hydrogel composition was analyzed using FTIR spectra, recorded on a Thermo-Scientific Nicolet 6700 FT-IR diamond crystal spectrometer, using the attenuated total reflectance (ATR) sampling technique FTIR spectra were recorded over the wavelength range of 700–4000 cm^−1^.

#### 2.3.2. Scanning Electron Microscopy (SEM)

Morphological analysis of the scaffolds was performed with SEM (Jeol JSM-7600 F). Samples, that were previously freeze-dried (using Martin Christ—Alpha 1–2 LDplus), were cut into slices, fixed on a holder using carbon tape, and sputtered with gold (using BAL-TEC SCD 005) and lyophilized in a vacuum chamber (VC 50 SalvisLab Vacucenter).

#### 2.3.3. Porosity Measurements

The porosity of hydrogels was determined by the solvent replacement method. Glycerol (*ρ* = 1.2038 g/cm^3^) was used as a wetting medium. Dried hydrogels were submerged in glycerol for 24 h, and weighed after removing excess glycerol from the surface:

(1) $$\text{Porosity}=\frac{\left(m_{\text{glycerol}}-m_{i}\right)}{\rho V}\times 100$$

where *m_i_* is the initial weight of the dry hydrogel, *m_glycerol_* is the weight of the hydrogel with glycerol, *ρ* is the density of glycerol, and *V* is the volume of the hydrogel sample.

#### 2.3.4. Mechanical Testing

Mechanical characteristics of the scaffolds were measured with a universal testing machine (Galdabini Quasar 50, Cardano al Campo, Italy) by the application of a uniaxial compression with 100-N load cell at room temperature. The Young’s modulus (E) was calculated from the linear part of the stress/strain curve and its final value is an average of three measurements.

#### 2.3.5. Water Contact Angle Measurement

The static water contact angle was measured using the sessile drop method by placing a drop (approximately 1 μL) of MilliQ water on the surface of the hydrogel. The measurements were performed using a contact angle meter Theta Lite-Biolin Scientific (with a measuring range of 0–180 deg. and accuracy ±0.1 deg., ±0.01 mN/m) equipped with the camera with 640 × 480 resolution and a maximum measuring speed of 60 fps. All measurements were repeated at least four times for each hydrogel.

#### 2.3.6. In Vitro Degradation Study

In vitro degradation study was conducted by immersion of the hydrogel samples in phosphate buffer (pH 7.40 at 37 °C). Samples were taken out of the buffer every two weeks, dried at 40 °C until constant mass, and weighed. Degradation was presented as remaining hydrogel mass percentage as a function of time:

(2) $$\text{Percent of remaining cryogel weight}=\frac{m_{t}}{m_{i}}\times 100$$

where *m_i_* is the initial weight of the dry hydrogel, and *m_t_* is the weight of the dried hydrogel sample at the time of measuring.

### 2.4. Biological Activity Studies

#### 2.4.1. Lactate Dehydrogenase Activity (LDH) Assay

Upon cell death, after contact with a cytotoxic material, LDH is released into the cell culture medium. LDH activity was determined as a measure for the cytotoxicity of the material and/or fabrication process in cell culture media after 2 days and for some groups after 2 and 28 days of cell culture according to the manufacturer’s kit instructions (Roche Diagnostics, Rotkreuz, Switzerland) and compared to LDH activity from cells on tissue culture plastic (TCP) in culture media (−control, assumed to be equal to 0% cell death) and cells cultured with 1% Triton X-100 (+control, assumed to be equal to 100% cell death). To overcome intergroup scaffold background interference, absorbance obtained from each scaffold type without cells was previously subtracted from each corresponding group. Values are presented as mean ± SD.

#### 2.4.2. Cell Expansion and Culture

Human mesenchymal stromal cells (hMSCs) were isolated from human bone marrow aspirate (Lonza) and characterized as previously described [60]. The cells were used at passage three and cultured under standard cell culture conditions (37 °C, 5% CO_2_) in an expansion medium consisting of Dulbecco’s modified Eagle medium (DMEM, 41,966,029 Gibco, Thermo Fisher Scientific, Waltham, MA, USA), 10% fetal bovine serum (FBS, 15,240,062 Gibco, Thermo Fischer Scientific), 1% nonessential amino acids (NEAA, 111,140,035 Gibco, Thermo Fischer Scientific), 1 ng/mL basic fibroblastic growth factor (bFGF, PHG0369 Gibco, Thermo Fischer Scientific), and 1% antibiotic–antimycotic (Anti–Anti, 15,240,062 Gibco, Thermo Fisher Scientific). After 7 days, cells were trypsinized and 1 × 10^6^ cells were seeded on each hydrogel by pipetting. After incubation for 90 min, 5 mL osteogenic media (DMEM, 10% FBS, 1% P/S/F, 50 μg/mL L-ascorbic acid (Sigma), 100 nM dexamethasone (Sigma) 10 mM beta-glycerolphosphate (Sigma)) was added. Constructs were cultured at 37 °C and 5% CO_2_ for up to 28 days and culture media was changed three times a week.

#### 2.4.3. Cell Adhesion to the Hydrogel Scaffolds

Cell adhesion was quantified by the number of attached cells using the Quant-iTTM PicoGreen^®^ dsDNA reagent kit (Invitrogen, Waltham, MA, USA). This allowed assessing the number of cells capable to adhere to the different hydrogel types in comparison to adhering to TCP. 16 h after cell seeding, the hydrogels were carefully washed twice in phosphate buffer saline (PBS, Medicago, Quebec City, QU, Canada) to wash away nonadherent cells. The remaining cells were lysed in 0.2% (*v*/*v*) Triton X-100 and 5 mM MgCl_2_ solution using two steel beads and a Mini BeadbeaterTM (Biospec, Bartlesville, OK, USA) three times at 25,000 RPM for 10 s each time. Samples were placed on ice between cycles for cooling. Acellular scaffolds were used as negative controls. After 48 h incubation at room temperature and centrifugation, the Quant-iTTM PicoGreen^®^ dsDNA reagent kit was used according to the manufacturer’s instructions. Fluorescence was read at an excitation wavelength of 480 nm and an emission wavelength of 520 nm with a plate reader (Tecan, Männedorf, Switzerland). The amount of DNA per sample was calculated according to the values of a DNA standard curve. DNA content of cells adhering to the scaffolds is presented relative to the DNA extracted from the same amount of cells seeded on TCP, which was set to 100%.

#### 2.4.4. AlamarBlue^®^ Assay

The AlamarBlue^®^ assay (Molecular Probes) was performed to get an indication of cell metabolic activity in cell culture supernatant on days 2, 7, and 28 days of the cell. Well plates containing the hydrogels were first washed once with PBS at 37 °C. The AlamarBlue^®^ assay was performed following the manufacturer’s instructions. Briefly, AlamarBlue^®^ solution was diluted at 1:10 in the control medium and 700 µL of the solution was added per well. Plates were then incubated for 70 min at 37 °C. For each sample, 100 µL of the sample was loaded into a 96-well black microtiter plate and fluorescence was measured at an excitation wavelength of 535 nm and an emission wavelength of 595 nm with a plate reader (TECAN).

#### 2.4.5. RNA Isolation and Real-Time PCR Analysis

Total mRNA was isolated using the Trizol reagent (Invitrogen) following the manufacturer’s protocol. Each hydrogel sample was broken down with steel balls and a Minibead Beater (Biospec) at 25,000 rpm for 10 s per cycle (6 cycles). The samples were kept on ice in between cycles. Total RNA (100 ng) was reverse transcribed to cDNA at 42 °C for 60 min with a High-Capacity RNA-to-cDNA kit (Applied Biosystems, Waltham, MA, USA) following the manufacturer’s protocol. The RNA was quantified using the Qubit 3.0 Fluorometer (Thermo Fisher Scientific) and the Qubit RNA HS Assay kit (Invitrogen). Real-time PCR (Biorad CFX96) was achieved with TaqMan probe detection (Applied Biosystems). Real-time PCR was performed for the housekeeping genes glyceraldehyde-3-phosphate dehydrogenase (GAPDH, Hs02758991_g1) and ACTB, Hs01060665_g1), and for the genes of interest collagen type I (COL1A2-I, Hs01028956_m1), alkaline phosphatase (ALPL, Hs01029144_m1), and osteocalcin (BGLAP, Hs01587814_g1). Each assay also contained a negative control without a cDNA template. Quantitative PCR data analysis was done by normalizing the target genes to the housekeeping gene glyceraldehyde-3-phosphate dehydrogenase (GAPDH), using qbase+ software, version 3.0 (Biogazelle, Zwijnaarde, Belgium—www.qbaseplus.com 15 July 2017).

#### 2.4.6. Statistics

Data are presented as mean values ± SD. For cell experiments, a Kolmogorov–Smirnov test was performed to test normality. Differences between groups were assessed by one-way ANOVA with Bonferroni correction or Kruskal–Wallis test depending on their distribution. SPSS program for Windows (Chicago, IL, USA) version 17.0 was used. Results were considered statistically significant at *p* values < 0.05.

## 3. Results and Discussion

**Scheme 1:**
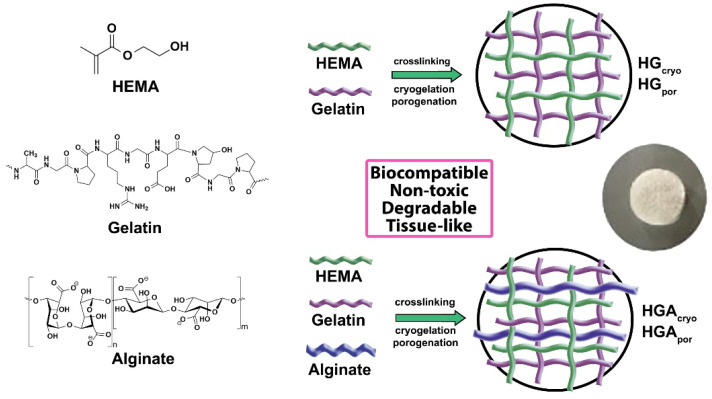
Synthesis path for interpenetrating polymeric networks and semi-interpenetrating polymeric networks hydrogel scaffolds made from 2-hydroxyethyl methacrylate, gelatin and alginate as an interpenetrant using cryogelation and porogenation methods.

Hydrogel scaffolds were successfully prepared using radical polymerization/crosslinking of interpenetrating polymeric networks consisting of synthetic 2-hydroxyethyl methacrylate (HEMA) and natural origin polymer gelatin, and using as an interpenetrant natural origin, linear polymer alginate, by cryogelation and porogenation methods (Scheme 1).

### 3.1. Structural Characteristics of Hydrogel Scaffolds

**Figure 1:**
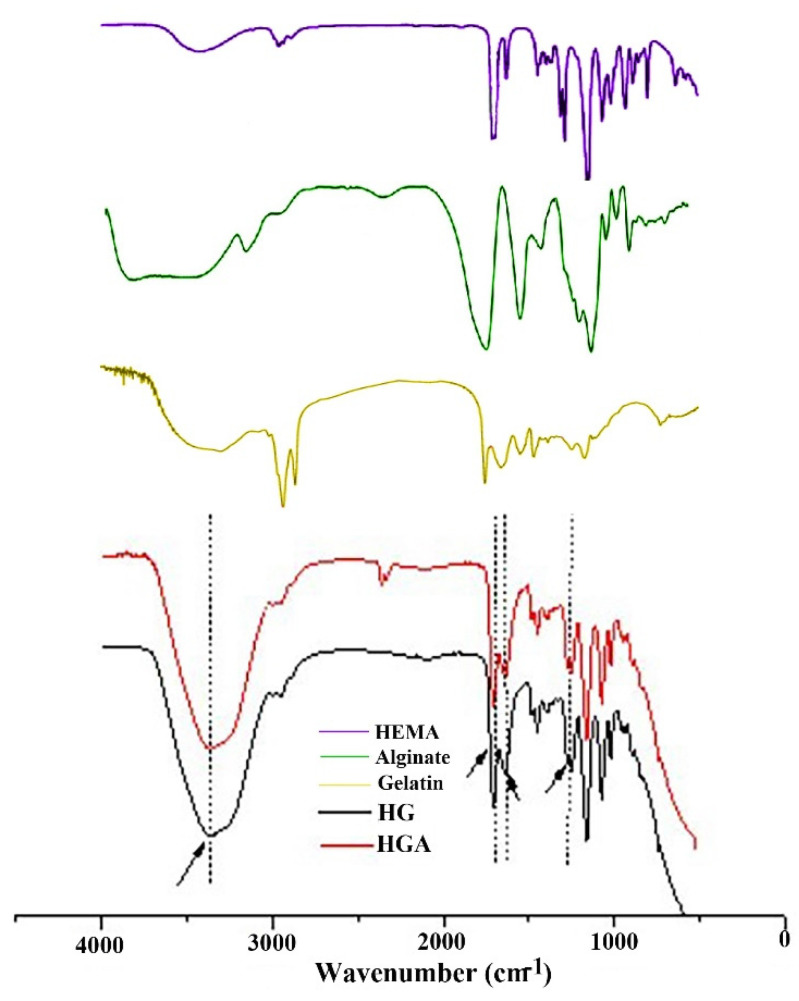
FTIR spectra of each component of scaffolds and hydrogel scaffolds.

Fourier transform infrared spectroscopy (FTIR) was used to reveal the structural characteristics of hydrogel scaffolds based on HEMA, gelatin, and alginate. FTIR spectra showed the characteristic bands of the functional groups that originated from hydrogel building components (HEMA, gelatin and alginate) (Figure 1). Peaks indicating the presence of HEMA are around 3360, 2940, 1710, and 1640 cm^−1^ and were designated to asymmetric and symmetric stretching vibrations (terminal hydroxyl group), methylene stretching, terminal vinyl, and a carbonyl group [61]. Peaks at 1640 and 1240 cm^−1^ were attributed to amide groups of gelatin [61,62]. Additionally, a peak at 1640 cm^−1^ can be assigned to asymmetric stretching of -COO^−^ of alginate [63,64]. There is no difference in the spectra obtained by the cryogelation and porogenation methods, which means that the method has no impact on the structural peculiarities of hydrogels (submarks are not given as _cryo_ and _por_ in Figure 1).

### 3.2. Hydrogel Scaffold Morphology

**Figure 2:**
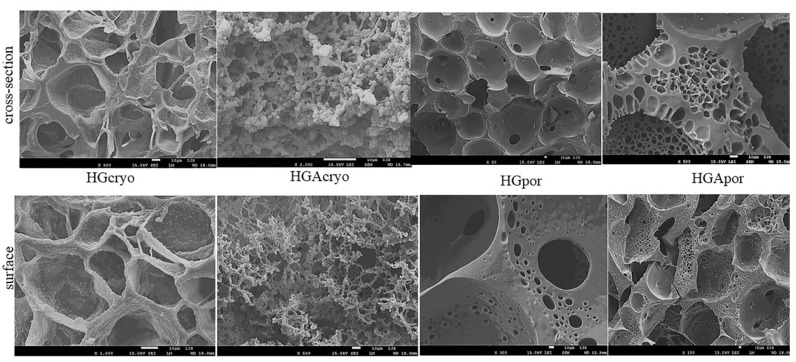
Representative SEM micrographs of the surface and a cross-section for HG_cryo_, HGA_cryo_, HGA_por_, HGA_por_ samples (scale bar: 10 μm).

An optimal scaffold for tissue regeneration engineering applications such as hard tissue regeneration must possess a highly porous structure with interconnected pores of at least 100 µm diameter for providing a large surface area that will allow cell penetration and ingrowth, uniform cell distribution and facilitates the neovascularization of the scaffolding construct [65,66]. The effects of the two different methods of synthesis as well as the scaffold’s composition on their morphology were examined by SEM. The obtained SEM micrographs of the cross-section (Figure 2) for the cryogels (HG and HGA) show uniformly distributed, highly porous structures with well-interconnected, ellipsoidal to spherical pores with a diameter of about 100 µm, which satisfy the criteria of scaffold morphology for hard tissue regeneration application. In comparison to scaffolds obtained by cryogelation, porogenated scaffolds exhibited different pattern morphology, more sharply defined pores mainly in spherical shapes with a fairly uniform distribution, probably due to the presence of a foam stabilizer which acts as “templates” during pore formation [67]. Furthermore, the cryogels also have higher porosity than porogenated scaffolds probably due to the sublimation of solvent crystals form well-interconnected large pores. Incorporating alginate as interpenetrant into scaffolds obtained by cryogelation showed a slight decrease in the porosity due to interpenetrated chains of alginate hindering the mobility and relaxation of the (HG) polymeric chains which decrease available free network space for solvent (water) absorption and resulting in lower content of solvent crystals (ice) and lower porosity after their sublimation. On the other hand, incorporating alginate as an interpenetrant into porogenated scaffolds leads to a slight increase in porosity probably because porogen (NaHCO_3_) and surfactant play a crucial role in the pore-forming process by porogenation.

The morphology of the scaffold surface was also examined by SEM owing to the scaffold surface being the initial and primary site of interaction with surrounding cells and tissue so scaffolds with a large and available surface area are favorable for tissue engineering application [65]. The obtained SEM micrographs of the scaffold surface (Figure 2) show different surface morphology which is following the cross-section morphology of the scaffolds. The surface of all the scaffolds is also a highly porous structure with well-interconnected pores while in porosity, the shape of pores and their distribution depends on the method used for hydrogel scaffold preparation and the presence of alginate chains as an interpenetrant. Scaffolds obtained by cryogelation have surfaces with higher porosity than porogenated scaffolds due to porogen agent effects during the pore-forming process.

### 3.3. Hydrogel Scaffold Porosity

The porosity data of the prepared hydrogel scaffolds are shown in Table 2. The cryogels possess higher porosity than porogenated scaffolds (cryosamples 84.25%, 82.26% and porogensamples 66.38%, 69.92%, respectively). There is an influence of the preparation method and alginate chains as an interpenetrant. As explained in the abovementioned discussion for morphological properties, alginate introduction into polymeric networks of HEMA and gelatin reduces porosity, for cryogel samples. Alginate-loaded porogen sample showed slightly higher porosity, which implies the influence of porogen on the distribution of alginate chains in a different way within HEMA and gelatin networks. The addition of alginate did not have a great impact on the porosity values, but it affected the size and wall thickness of the pores. In sample HGApor, the addition of alginate resulted in highly interconnected smaller pores with a high fraction of very small pores in the wall of large pores. It can be noticed that scaffolds obtained by cryogelation are more favorable for tissue regeneration [68].

### 3.4. Hydrophilicity of Hydrogel Scaffolds

**Scheme 2:**
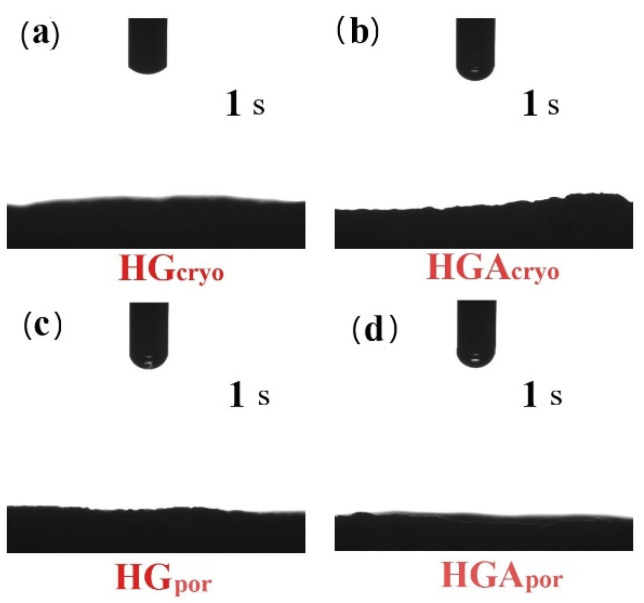
Hydrophilicity of hydrogel scaffolds.

The obtained results of contact angle measurements performed at 0 and 1 s revealed that the surfaces of all scaffolds HG_cryo_, HGA_cryo_, HG_por_ and HGA_por_ are fully hydrophilic (Scheme 2a–d). Water completely wetted scaffolds surfaces and drop immediately disappeared after putting on their surface confirming the scaffolds as favorable biomaterials for adhesion, proliferation, and differentiation of various types of cells. This behavior was expected due to the high hydrophilicity of both natural origin polymers, gelatin, and alginate, which resulted in the high content of hydrophilic groups in the HG and HGA scaffolds surface [69,70]. It can be noted that both cryogelation and porogenation methods used to synthesize scaffolds made of polymers of synthetic and natural origin proved to be successful in making fully hydrophilic scaffolds. Surface hydrophilicity of the biomaterials is an influential parameter that controls in vivo tissue compatibility as well as in vivo functionality and safety of many biomaterials/scaffolds because the surface of scaffolds is the initial and primary contact of interaction with cells and tissues [71]. Hydrophilic surface modifications of biomaterials are widely considered to enhance cell adhesion and activity compared to more hydrophobic counterparts [72]. The surface hydrophilicity depends on many factors such as surface morphology and roughness, porosity, surface phase separation structure, chemical composition as well as the nature of the monomeric/polymeric components. Herein, we confirmed that both the preparation method and the composition of hydrogel scaffolds were tuned to obtain fully hydrophilic samples.

### 3.5. Mechanical Properties of Hydrogel Scaffolds

The key factor of tissue regenerative engineering is to create ECM-like scaffolding biomaterials with desired optimal mechanical strength to ensure mechanical and shape stability and biomechanical stimulation of cells to form effective engineered tissues. Mechanical properties of HEMA/gelatin/alginate-based scaffolds are expressed through Young’s modulus. The values for Young’s modulus (E) obtained (Table 2) show dependence on the preparation method and loading of alginate as an interpenetrant. The highest E value was obtained for HGA_cryo_ sample (9.75 MPa). E value for cryosamples (HG_cryo_ and HGA_cryo_) increase four times more when alginate is loaded as an intepenetrant into HEMA and gelatin polymeric networks. Sample loaded with alginate obtained by porogenation show decreased E value (HGA_por_ sample showed about four times lower value than HG_por_ sample). When considering the progenation method, loading alginate within the HEMA and gelatin networks leads to the change of morphological pattern and significantly lowers the mechanical strength of the sample.

### 3.6. In Vitro Degradation Behavior of Hydrogel Scaffolds

Designing the biologically active scaffold with optimal degradation behavior is a very important fact for successfully engineered tissues. An optimal biodegradable scaffold as an indispensable element for engineering living tissues should be able to degrade with time in vivo, at a controlled degradation rate that guarantees the necessary mechanical support until the regeneration process is successfully finished [73]. The degradation of HG and HGA scaffolds obtained by cryogelation and porogenation, was evaluated in vitro by immersing samples in a phosphate buffer (pH 7.40 at 37 °C) for 16 weeks. The obtained results were presented as a percentage of their weight loss (Table 2). These two scaffold types exhibited different degradation behavior.

It can be said that scaffolds obtained by cryogelation showed higher mass loss values for the same period of degradation, 3.65% and 5.04%, compared to scaffolds obtained by porogenation which exhibited lower values of mass loss, 2.28% and 1.59%. This behavior can be elucidated by higher porosity of cryosample and alginate hydrophilicity which facilitates faster and easier water penetration inside the scaffolds and a higher degree of degradation. Porogen samples possess lower porosity therefore water penetrates slowly within the scaffold and the degree of degradation is lower. This degradation behavior indicates the influence of the preparation method on the degradability of the hydrogel scaffolds, as well as the presence of alginate as an interpenetrant.

### 3.7. Cytotoxicity of Hydrogel Scaffolds: Lactate Dehydrogenase Activity (LDH) Assay

**Figure 3:**
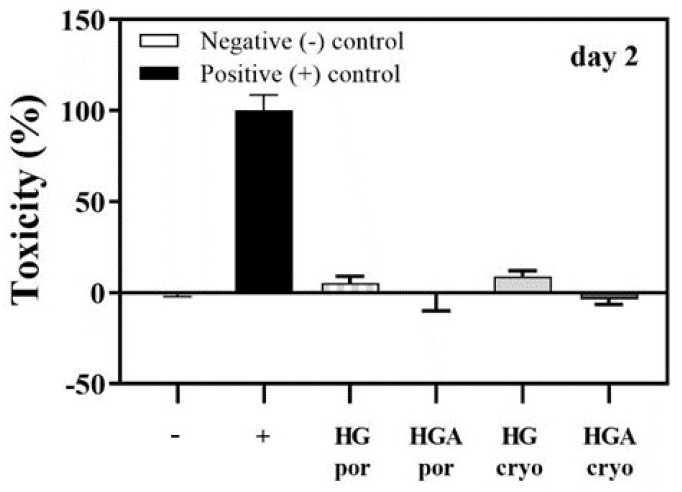
Cell viability based on LDH activity after 2 days of culture cells on HG_por_, HGA_por_, HG_cryo_, or HGA_cryo_ hydrogel scaffolds. Negative control (0% cell death), positive control (100% cell death). Values represent the mean ± SD.

The LDH assay was used as an index of cell death to assess the preliminary cytotoxicity of the hydrogel scaffolds (HG_por_ and HGA_por_, HG_cryo_, HGA_cryo_) (Figure 3). Results are presented relative to LDH activity from cells on tissue culture plastic in culture media (−control) and cells cultured with 1% Triton (+control). Alginate and gelatin are two natural and biocompatible polymers widely used in biomedical applications and tissue engineering. In addition, HEMA has been proven to be biocompatible [74]. Since LDH activities below 30% are considered biologically not relevant, it can be concluded that all hydrogel scaffold materials show favorable cytocompatibility. In addition, there were no significant differences between scaffolds generated via porogenation or cryogelation. Similarly, there were no significant differences between scaffolds containing alginate or not. These results suggest that both methods used to create hydrogel scaffolds and the inclusion of alginate as an interpenetrant did not negatively affect cell viability.

Given the good cytocompatibility and their versatility important for tissue regeneration (degradability, hydrophilicity, morphological, and mechanical), the cryogelated scaffolds HG_cryo_ and HGA_cryo_ were selected and further loaded with apatite (HAp) [75] to perform more biological activity studies. Morphological, mechanical properties, hydrophilicity, degradation, and in vivo biocompatible properties (zebrafish model) of HG_cryo_/HAp and HGA_cryo_/HAp were performed [76].

### 3.8. Influence of Hydrogel Scaffold Properties on Cell Behaviour

#### 3.8.1. Cell Adhesion to the Hydrogel Scaffolds

**Figure 4:**
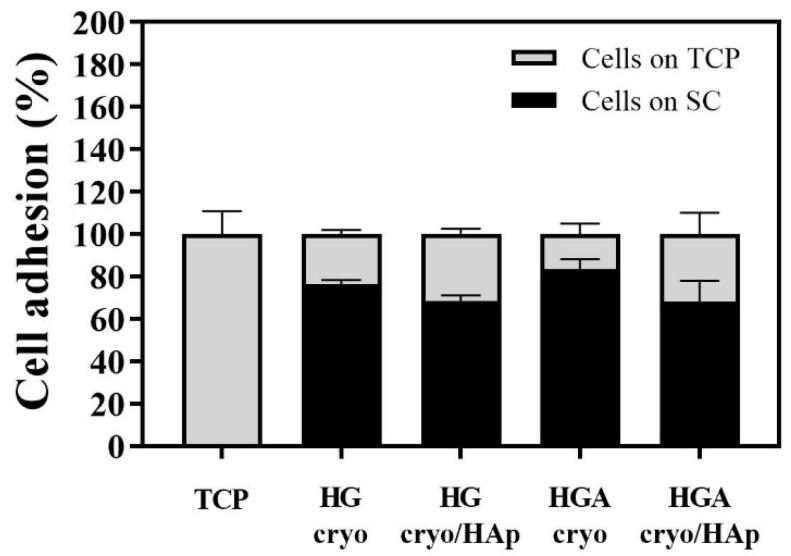
hMSC adhesion on day 1 after seeding. A majority of the seeded cells were able to adhere to the scaffolds with or without HAp (HG_cryo_, HG_cryo_/HAp, HGA_cryo_, HGA_cryo_/HAp) 1 day after seeding.

Adherent cell behavior, with a biomaterial surface depends on chemical composition, mechanical characteristics, wettability, and morphology [77,78,79]. A DNA assay was performed to quantify the number of adhered cells to the different hydrogel scaffolds on day 1 (Figure 4). A majority of the hMSCs (on average between 68% and 82% of the cells in comparison to TCP) were able to adhere to the scaffold samples. The gelatin component of HG and HGA scaffolds, a natural macromolecular hydrophilic material [80] might have been supportive for cell adhesion through their arginine-glycine-aspartic acid (RGD) peptide sequence. Furthermore, similar cell adhesion percentages were obtained in HG and HGA scaffolds, suggesting that the use of alginate as an interpenetrant did not interfere with the cell adhesion behavior. Interestingly, despite HAp having been reported to support cell adhesion through DNA and protein binding [81], no significant differences were observed between scaffolds containing HAp or not. These results suggest that both HG and HGA scaffolds provide chemical cues that support the adhesion of osteoblastic progenitor cells.

#### 3.8.2. Cytotoxicity of Cryogelated Scaffolds with or without HAp on Seeded Cells

**Figure 5:**
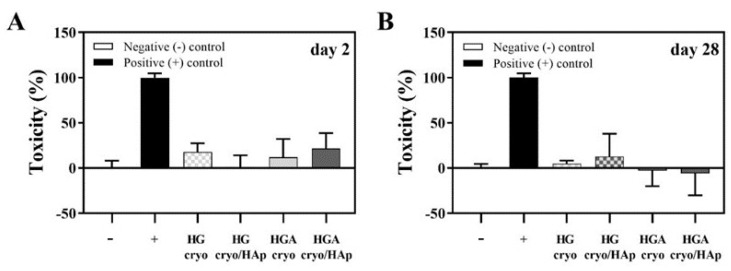
Cytotoxicity of scaffolds (HG_cryo_, HG_cryo_/HAp, HGA_cryo_, HGA_cryo_/HAp) on seeded hMSCs as determined by LDH activity in culture media collected after 2 (**A**) or 28 days (**B**) days. Values represent mean ± SD (*n* = 4).

We next investigated the cytocompatibility of the cryogelated scaffolds (HG_cryo_, HG_cryo_/HAp, HGA_cryo_, HGA_cryo_/HAp) with an LDH. It was noticed that the inclusion of HAp did not affect the cells in a negative way after 2 and 28 days (Figure 5). All average LDH activity values remained below 30%. It is well accepted that HAp is well tolerated with no adverse local or systemic toxicity in vivo [82]. In agreement, our in vitro studies show that HAp loading did not negatively affect the cells seeded onto any of the scaffolds after 2 or 28 days of cell culture.

#### 3.8.3. Cell Metabolic Activity

**Figure 6:**
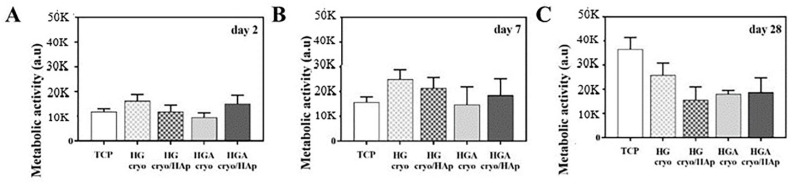
Alamar blue assay. Determination of the total cell metabolic activity after culture cells for 2 (**A**), 7 (**B**), and 28 days (**C**) on scaffolds (HG_cryo_, HG_cryo_/HAp, HGA_cryo_, HGA_cryo_/HAp). Values represent the mean ± SD (*n* = 4/group).

The metabolic activity of hMSCs on the cryogelated hydrogel scaffolds (HG_cryo_, HG_cryo_/HAp, HGA_cryo_, HGA_cryo_/HAp) was determined with an AlamarBlue^®^ assay on days 2, 7, and 28 after seeding the cells (Figure 6A–C). This assay can give insight into cell metabolic activity and/or cell proliferation [83,84]. There was no significant difference in cell metabolic activity between the groups at the assessed time points.

#### 3.8.4. Expression of Osteogenesis-Related Genes

**Figure 7:**
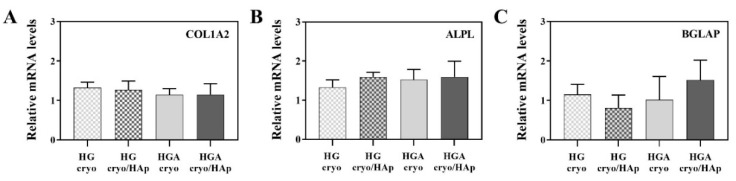
Gene expression levels of collagen type 1 (COL1A2) (**A**), alkaline phosphatase (ALPL) (**B**), and osteocalcin (BGLAP) (**C**) in hMSCs that were cultured for 28 days in an osteogenic medium on the cryogelated scaffolds (HG_cryo_, HG_cryo_/HAp, HGA_cryo_, HGA_cryo_/HAp) show no differences between the groups. Values were normalized to GAPDH as the housekeeping gene and are expressed as fold change relative to the average across all groups, which were set to 1. Values represent the mean ± SD (*n* = 3/group).

We further looked at the influence of HAp and alginate interpenetrant on gene expression during osteogenic differentiation. The hMSCs seeded onto the scaffolds (HG_cryo_, HG_cryo_/HAp, HGA_cryo_, HGA_cryo_/HAp) were cultivated in an osteogenic differentiation medium for 4 weeks (Figure 7). After 4 weeks, the gene expression levels indicative for extracellular collagen type 1 protein (COL1A2), osteogenic differentiation marker alkaline phosphatase (ALPL), and ECM protein osteocalcin (BGLAP) were compared between the groups. There was no significant difference between the tested materials—all scaffolds performed equally in terms of osteoconductivity. Biocompatibility and osteoconductivity of HAp are generally accepted [85]. Here we hypothesize that the apatite particles appeared to be less effective in stimulating osteogenic differentiation of hMSCs probably due to events in the scaffold’s microenvironment during the regeneration process.

## 4. Conclusions

New cryogelated and porogenated bioactive HEMA/gelatin/alginate scaffolding biomaterials with incorporated HAp were successfully designed for tissue regeneration. Our guiding idea was validated through the “replica” of the interpenetrated network structure of the native extracellular matrix. The effects of preparation methods and adding alginate as an interpenetrant in HEMA and gelatin polymeric networks on hydrogel scaffold’s properties were confirmed. The hydrogel scaffold’s structural characteristics were confirmed using FTIR spectra analysis. Morphological patterns are favorable interconnected pore structures dependent on alginate presence and method preparation. Porosity is in the range of 66.38–84.25%. Young modulus (mechanical properties) values are in the range of 0.99–9.75 MPa. In vitro degradation studies were followed for a period of 16 weeks and show 1.59–5.04% mass percent loss. Cell viability assay showed excellent biocompatible behavior. Cryogelated scaffolds were shown to possess superior functionalities essential for tissue regeneration: fully hydrophilicity, degradability, mechanical properties, and favorable biocompatibility. Furthermore, cryogelated scaffolds loaded with apatite showed a good cell adhesion capacity, biocompatibility and non-toxic behavior. All scaffolds performed equally in terms of metabolic activity and osteoconductivity. Overall, cryogelated scaffolds with/without HAp could represent a new venue to promote osteoconductivity and enhance hard tissue repair.

## Figures and Tables

**Table 1:** Composition and marks of the hydrogel scaffold samples.

Sample	Component 1	Component 2	Component 3	Cross-Linker for HEMA	Cross-Linker for Gelatin	Initiator/Activator	Pore Formation Method
HG_cryo_	HEMA	Gelatin	-	EGDMA	EDC	PPS/TEMED	Cryogelation
HGA_cryo_	HEMA	Gelatin	Alginate	EGDMA	EDC	PPS/TEMED	Cryogelation
HG_por_	HEMA	Gelatin	-	EGDMA	EDC	PPS/TEMED	Porogenation
HGA_por_	HEMA	Gelatin	Alginate	EGDMA	EDC	PPS/TEMED	Porogenation

**Table 2:** Values of porosity, Young’s modulus, and percentage of degradation for hydrogel scaffolds.

Sample	Porosity (%)	Young’s Modulus (MPa)	Mass Loss (%)
HG_cryo_	84.25 ± 3.58	2.08 ± 0.08	3.65 ± 0.10
HGA_cryo_	82.26 ± 3.51	9.75 ± 0.24	5.04 ± 0.12
HG_por_	66.38 ± 3.02	4.61 ± 0.15	2.28 ± 0.07
HGA_por_	69.92 ± 3.14	0.99 ± 0.04	1.59 ± 0.03

## Data Availability

Not applicable.

## References

[1] Gavrilov L.A., Gavrilova N.S. (2015). New developments in the biodemography of aging and longevity. Gerontology.

[2] Geris L., Papantoniou I. (2019). The third era of tissue engineering: Reversing the innovation drivers. Tissue Eng.-Part A.

[3] World Health Organization (2020). WHO Methods and Data Sources for Global Burden of Disease Estimates 2000–2019.

[4] Geckil H., Xu F., Zhang X., Moon S., Demirci U. (2010). Engineering hydrogels as extracellular matrix mimics. Nanomedicine.

[5] Kim T.G., Shin H., Lim D.W. (2012). Biomimetic scaffolds for tissue engineering. Adv. Funct. Mater..

[6] Ahmed E.M. (2015). Hydrogel: Preparation, characterization, and applications: A review. J. Adv. Res..

[7] Maitra J., Shukla V.K. (2014). Cross-linking in hydrogels-A review. Am. J. Polym. Sci..

[8] Fan C., Wang D.-A. (2017). Macroporous hydrogel scaffolds for three-dimensional cell culture and tissue engineering. Tissue Eng. Part B Rev..

[9] Eltom A., Zhong G., Muhammad A. (2019). Scaffold techniques and designs in tissue engineering functions and purposes: A review. Adv. Mater. Sci. Eng..

[10] Zhang Y.S., Khademhosseini A. (2017). Advances in engineering hydrogels. Science.

[11] Yahia L., Chirani N., Yahia L., Gritsch L., Motta F., Chirani S., Fare S. (2015). History and applications of hydrogels. J. Biomed. Sci..

[12] Bustamante-Torres M., Romero-Fierro D., Arcentales-Vera B., Palomino K., Magaña H., Bucio E. (2021). Hydrogels classification according to the physical or chemical interactions and as stimuli-sensitive materials. Gels.

[13] Langer R., Vacanti J.P. (1993). Tissue Engineering. Science.

[14] O’Brien F.J. (2011). Biomaterials & scaffolds for tissue engineering. Mater. Today.

[15] Hunt J.A., Chen R., van Veen T., Bryan N. (2014). Hydrogels for tissue engineering and regenerative medicine. J. Mater. Chem. B.

[16] Zhu J., Marchant R.E. (2011). Design properties of hydrogel tissue-engineering scaffolds. Expert Rev. Med. Devices.

[17] Wang L., Neumann M., Fu T., Li W., Cheng X., Su B.L. (2018). Porous and responsive hydrogels for cell therapy. Curr. Opin. Colloid Interface Sci..

[18] Slaughter B.V., Khurshid S.S., Fisher O.Z., Khademhosseini A., Peppas N.A. (2009). Hydrogels in regenerative medicine. Adv. Mater..

[19] El-Sherbiny I.M., Yacoub M.H. (2013). Hydrogel scaffolds for tissue engineering: Progress and challenges. Glob. Cardiol. Sci. Pract..

[20] Zhang J., Griesbach J., Ganeyev M., Zehnder A.-K., Zeng P., Schädli G.N., de Leeuw A., Lai Y., Rubert M., Müller R. (2022). Long-term mechanical loading is required for the formation of 3D bioprinted functional osteocyte bone organoids. Biofabrication.

[21] Zhang J., Eyisoylu X., Qin X.-H., Rubert M., Müller R. (2021). 3D bioprinting of graphene oxide-incorporated cell-laden bone mimicking scaffolds for promoting scaffold fidelity, osteogenic differentiation and mineralization. Acta Biomater..

[22] Urruela-Barrios R., Ramírez-Cedillo E., de León A.D., Alvarez A., Ortega-Lara W. (2019). Alginate/gelatin hydrogels reinforced with TiO_2_ and β-TCP fabricated by microextrusion-based printing for tissue regeneration. Polymers.

[23] Boccaccini A., Gough J. (2007). Tissue Engineering Using Ceramics and Polymers.

[24] Bhatia S. (2016). Natural Polymer Drug Delivery Systems.

[25] Kumbar S., Laurencin C., Deng M. (2014). Natural and Synthetic Biomedical Polymers.

[26] Sarker B., Li W., Zheng K., Detsch R., Boccaccini A.R. (2016). Designing porous bone tissue engineering scaffolds with enhanced mechanical properties from composite hydrogels composed of modified alginate, gelatin and bioactive glass. ACS Biomater. Sci. Eng..

[27] Harini B., Shadamarshan R.P.K., Rao S.H., Selvamurugan N., Balagangadharan K. (2017). Natural and synthetic polymers/bioceramics/bioactive compounds-mediated cell signalling in bone tissue engineering. Int. J. Biol. Macromol..

[28] Klotz B.J., Gawlitta D., Rosenberg A.J., Malda J., Melchels F.P. (2016). Gelatin-methacryloyl hydrogels: Towards biofabrication-based tissue repair. Trends Biotechnol..

[29] Yue K., Trujillo-de Santiago G., Alvarez M.M., Tamayol A., Annabi N., Khademhosseini A. (2015). Synthesis, properties, and biomedical applications of gelatin methacryloyl (GelMA) hydrogels. Biomaterials.

[30] Sun M., Sun X., Wang Z., Guo S., Yu G., Yang H. (2018). Synthesis and properties of gelatin methacryloyl (GelMA) hydrogels and their recent applications in load-bearing tissue. Polymers.

[31] Zheng J., Zhao F., Zhang W., Mo Y., Zeng L., Li X. (2018). Sequentially-crosslinked biomimetic bioactive glass/gelatin methacryloyl composites hydrogels for bone regeneration. Mater. Sci. Eng. C.

[32] Rottensteiner U., Sarker B., Heusinger D., Dafinova D., Rath S.N., Beier J.P., Kneser U., Horch R.E., Detsch R., Boccaccini A.R. (2014). In vitro and in vivo biocompatibility of alginate dialdehyde/gelatin hydrogels with and without nanoscaled bioactive glass for bone tissue engineering applications. Materials.

[33] Sarker B., Zehnder T., Rath S.N., Horch R.E., Kneser U., Detsch R., Boccaccini A.R. (2017). Oxidized alginate-gelatin hydrogel: A favorable matrix for growth and osteogenic differentiation of adipose-derived stem cells in 3D. ACS Biomater. Sci. Eng..

[34] Zhang C., Wang W., Zhao X., Wang H., Yin H. (2020). Preparation of alginate oligosaccharides and their biological activities in plants: A review. Carbohydr. Res..

[35] Lee K.Y., Mooney D.J. (2012). Alginate: Properties and biomedical applications. Prog. Polym. Sci..

[36] Sun J., Tan H. (2013). Alginate-based biomaterials for regenerative medicine applications. Materials.

[37] Zhong H., Gao X., Cheng C., Liu C., Wang Q., Han X. (2020). The Structural Characteristics of Seaweed Polysaccharides and Their Application in Gel Drug Delivery Systems. Mar. Drugs.

[38] Hernandez-Gonzalez A.C., Tellez-Jurado L., Rodriguez-Lorenzo L.M. (2020). Alginate hydrogels for bone tissue engineering, from injectables to bioprinting: A review. Carbohydr. Polym..

[39] Uyen N.T.T., Hamid Z.A.A., Tram N.X.T., Ahmad N. (2020). Fabrication of alginate microspheres for drug delivery: A review. Int. J. Biol. Macromol..

[40] Saltz A., Kandalam U. (2016). Mesenchymal stem cells and alginate microcarriers for craniofacial bone tissue engineering: A review. J. Biomed. Mater. Res. A.

[41] Szekalska M., Puciłowska A., Szymańska E., Ciosek P., Winnicka K. (2016). Alginate: Current use and future perspectives in pharmaceutical and biomedical applications. Int. J. Polym. Sci..

[42] Schrieber R., Gareis H. (2007). Gelatine Handbook: Theory and Industrial Practice.

[43] Echave M.C., Saenz del Burgo L., Pedraz J.L., Orive G. (2017). Gelatin as biomaterial for tissue engineering. Curr. Pharm. Des..

[44] Hoque M.E., Nuge T., Tshai K.Y., Nordin N., Prasad V. (2015). Gelatin based scaffolds for tissue engineering-a review. Polym Res. J..

[45] Bello A.B., Kim D., Kim D., Park H., Lee S.-H. (2020). Engineering and functionalization of gelatin biomaterials: From cell culture to medical applications. Tissue Eng. Part B Rev..

[46] Djagny K.B., Wang Z., Xu S. (2001). Gelatin: A valuable protein for food and pharmaceutical industries: Review. Crit. Rev. Food Sci. Nutr..

[47] Davidenko N., Schuster C.F., Bax D.V., Farndale R.W., Hamaia S., Best S.M., Cameron R.E. (2016). Evaluation of cell binding to collagen and gelatin: A study of the effect of 2D and 3D architecture and surface chemistry. J. Mater. Sci. Mater. Med..

[48] Afewerki S., Sheikhi A., Kannan S., Ahadian S., Khademhossein A. (2019). Gelatin-polysaccharide composite scaffolds for 3D cell culture and tissue engineering: Towards natural therapeutic. Bioeng.Transl. Med..

[49] Horák D., Pethrick R.A., Zaikov G.E., Horák D. (2007). Application of poly(2-hydroxyethyl methacrylate) in medicine. Polymers and Composites: Synthesis, Properties, and Applications, Polymer Yearbook.

[50] Kopeček J. (2009). Hydrogels from soft contact lenses and implants to self-assembled nanomaterials. J. Polym. Sci. A.

[51] Park S., Nam S.H., Koh W.-G. (2012). Preparation of collagen-immobilized poly(ethylene glycol)/poly(2-hydroxyethyl methacrylate) interpenetrating network hydrogels for potential application of artificial cornea. J. Appl. Polym. Sci..

[52] Hidzir N.M., Radzali N.A.M., Rahman I.A., Shamsudin S.A. (2020). Gamma irradiation-induced grafting of 2-hydroxyethyl methacrylate (HEMA) onto ePTFE for implant applications. Nucl. Eng. Technol..

[53] Passos M.F., Dias D.R.C., Bastos G.N.T., Jardini A.L., Benatti A.C.B., Dias C.G.B.T., Filho R.M. (2016). pHEMA hydrogels: Synthesis, kinetics and in vitro tests. J. Therm. Anal. Calorim..

[54] Dobić S.N., Filipović J.M., Tomić S.Lj. (2012). Synthesis and characterization of poly(2-hydroxyethyl methacrylate/itaconic acid)/poly(ethyleneglycol dimethacrylate) hydrogels. Chem. Eng. J..

[55] Filipović V.V., Bozić Nedeljković B.D., Vukomanović M., Tomić S.Lj. (2018). Biocompatible and degradable scaffolds based on 2-hydroxyethyl methacrylate, gelatin and poly(beta amino ester) crosslinkers. Polym. Test..

[56] Filipović V.V., Babić Radić M.M., Vuković J.S., Vukomanović M., Rubert M., Hofmann S., Müller R., Tomić S.Lj. (2022). Biodegradable hydrogel scaffolds based on 2-hydroxyethyl methacrylate, gelatin, poly(β-amino esters), and hydroxyapatite. Polymers.

[57] Takić Miladinov D., Tomić S., Stojanović S., Najdanović J., Filipović J., Trajanović M., Najman S. (2016). Synthesis, Swelling properties and evaluation of genotoxicity of hydrogels based on (meth)acrylates and itaconic acid. Mater. Res..

[58] Jodati H., Yılmaz B., Evis Z. (2020). A review of bioceramic porous scaffolds for hard tissue applications: Effects of structural features. Ceram. Int..

[59] Jeong J., Kim J.H., Shim J.H., Hwang N.S., Heo C.Y. (2019). Bioactive calcium phosphate materials and applications in bone regeneration. Biomater. Res..

[60] Hofmann S., Hagenmüller H., Koch A.M., Müller R., Vunjak-Novakovic G., Kaplan D.L., Merkle H.P., Meinel L. (2007). Control of in vitro tissue-engineered bone-like structures using human mesenchymal stem cells and porous silk scaffolds. Biomaterials.

[61] Tomić S.Lj., Babić M.M., Antić K.M., Vuković J.S., Malešić N.B., Filipović J.M. (2014). pH-sensitive hydrogels based on (meth)acrylates and itaconic acid. Macromol. Res..

[62] Li Y., Zhang X., Zhao Y., Ding J., Lin S. (2018). Investigation on complex coacervation between fish skin gelatin from cold-water fish and gum arabic: Phase behavior, thermodynamic, and structural properties. Food Res. Int..

[63] Wang Q.-Q., Liu Y., Zhang C.-J., Zhang C., Zhu P. (2019). Alginate/gelatin blended hydrogel fibers cross-linked by Ca^2+^ and oxidized starch: Preparation and properties. Mater. Sci. Eng. C.

[64] Vieira E.F., Cestari A.R., Airoldi C., Loh W. (2009). Polysaccharide-based hydrogels: Preparation, characterization, and drug interaction behavior. Biomacromolecules.

[65] Dhandayuthapani B., Yoshida Y., Maekawa T., Sakthi Kumar D. (2011). Polymeric scaffolds in tissue engineering application: A review. Int. J. Polym. Sci..

[66] Leon C.A. (1998). New perspectives in mercury porosimetry. Adv. Colloid Interface Sci..

[67] Rill R.L., Van Winkle D.H., Locke B.R. (1998). Templated pores in hydrogels for improved size selectivity in Gel Permeation Chromatography. Anal. Chem..

[68] Collins M.N., Ren G., Young K., Pina S., Reis R.L., Oliveira J.M. (2021). Scaffold fabrication technologies and structure/function properties in bone tissue engineering. Adv. Funct. Mater..

[69] Guo S., Zhu X., Li M., Shi L., Ong J.L.T., Jańczewski D., Neoh K.G. (2016). Parallel control over surface charge and wettability using polyelectrolyte architecture: Effect on protein adsorption and cell adhesion. ACS Appl. Mater. Interf..

[70] Ayala R., Zhang C., Yang D., Hwang Y., Aung A., Shroff S.S., Arce F.T., Lal R., Arya G., Varghese S. (2011). Engineering the cell–material interface for controlling stem cell adhesion, migration, and differentiation. Biomaterials.

[71] Idaszek J., Kijeńska E., Łojkowski M., Swieszkowski W. (2016). How important are scaffolds and their surface properties in regenerative medicine. Appl. Surf. Sci..

[72] Kyriakides T.R., Badylak S.F. (2015). Molecular events at tissue/biomaterial interface. Host Response to Biomaterials.

[73] Derby B. (2012). Printing and prototyping of tissues and scaffolds. Science.

[74] Prasitsilp M., Siriwittayakorn T., Molloy R., Suebsanit N., Siriwittayakorn P., Veeranondha S. (2003). Cytotoxicity study of homopolymers and copolymers of 2-hydroxyethyl methacrylate and some alkyl acrylates for potential use as temporary skin substitutes. J. Mater. Sci. Mater. Med..

[75] Kurtjak M., Vukomanović M., Krajnc A., Kramer L., Turk B., Suvorov D. (2016). Designing Ga(III)-containing hydroxyapatite with antibacterial activity. RSC Adv..

[76] Tomić S.Lj., Nikodinović-Runić J., Vukomanović M., Babić M.M., Vuković J.S. (2021). Novel hydrogel scaffolds based on alginate, gelatin, 2-hydroxyethyl methacrylate, and hydroxyapatite. Polymers.

[77] Rahmati M., Silva E.A., Reseland J.E., Heyward C.A., Haugen H.J. (2020). Biological responses to physicochemical properties of biomaterial surface. Chem. Soc. Rev..

[78] Chen L., Yan C., Zheng Z. (2018). Functional polymer surfaces for controlling cell behaviors. Mater. Today.

[79] Kołbuk D., Heljak M., Choinska E., Urbanek O. (2020). Novel 3D hybrid nanofiber scaffolds for bone regeneration. Polymers.

[80] Wang H.M., Yang Z. (2012). Short-peptide-based molecular hydrogels: Novel gelation strategies and applications for tissue engineering and drug delivery. Nanoscale.

[81] Han Y., Li S., Cao X., Yuan L., Wang Y., Yin Y., Qiu T., Dai H., Wang X. (2014). Different inhibitory effect and mechanism of hydroxyapatite nanoparticles on normal cells and cancer cells in vitro and in vivo. Sci. Rep..

[82] Kattimani V.S., Kondaka S., Lingamaneni K.P. (2016). Hydroxyapatite–Past, Present, and Future in Bone Regeneration. Bone Tissue Regen. Insights.

[83] Fields R.D., Lancaster M.V. (1993). Dual-attribute continuous monitoring of cell proliferation/cytotoxicity. Am. Biotechnol. Lab..

[84] Ahmed S.A., Gogal R.M., Walsh J.E. (1994). A new rapid and simple non-radioactive assay to monitor and determine the proliferation of lymphocytes: An alternative to [3H] thymidine incorporation assay. J. Immunol. Methods.

[85] Yang X., Li Y., Liu X., Zhang R., Feng Q. (2018). In Vitro Uptake of Hydroxyapatite Nanoparticles and Their Effect on Osteogenic Differentiation of Human Mesenchymal Stem Cells. Stem Cells Int..

